# Lipoxins: A Novel Regulator in Embryo Implantation

**DOI:** 10.1100/tsw.2011.15

**Published:** 2011-01-18

**Authors:** Jing Xiong, Pan Zeng, Duyun Ye

**Affiliations:** ^1^ Institute of Pathology, Tongji Hospital, Tongji Medical College, Huazhong University of Science and Technology, Wuhan, China; ^2^ Department of Pathophysiology, Tongji Medical College, Huazhong University of Science and Technology, Wuhan, China

**Keywords:** lipoxins, inflammation, implantation

## Abstract

Embryo implantation is essential for mammalian pregnancy, which involves intricate cross-talk between the blastocyst and the maternal endometrium. Recent advances have identified various molecules crucial to implantation and endometrial receptivity, including leukemia inhibitory factor, calcitonin, and homeobox A10. There is a close relationship between implantation and inflammation. Lipoxins, important in the resolution of inflammation, may be a potential regulator in implantation. Here we discuss the hypothesis that lipoxins may work as a novel regulator in embryo implantation and the possible molecular mechanisms.

## EMBRYO IMPLANTATION

Although many underlying causes of human infertility have been overcome by a variety of assisted reproductive techniques, such as *in vitro* fertilization and embryo transfer (IVF/ET) programs, pregnancy rates remain disappointingly low[1] owing to implantation failure. Therefore, embryo implantation is essential for pregnancy and remains a problem to be solved in reproductive medicine. Understanding the mechanism underlying implantation will help to improve assisted reproductive technology and develop novel contraceptives. Embryo implantation is a basic physiological process. Many of the features and signaling pathways involved in implantation are also related to some pathological processes, such as inflammation and tumors. Therefore, understanding the intricacy of implantation might help to better understand the complexities of these pathological processes and provide valuable insights into other fields.

Embryo implantation consists of a series of sequential biological processes in which the blastocyst apposes and adheres to the wall of the uterus, and subsequently invades it. Complex cross-talk between the blastocyst and the maternal endometrium is implicated in the process of implantation. Implantation can only take place during a very specific, short time in the mid-secretory phase of the menstrual cycle (approximately 7–10 days after ovulation[2]), commonly known as the window of implantation (WOI). During this period, the uterus undergoes a sequence of cellular and molecular events in the endometrium and differentiates into a state of receptivity. Following extensive research into embryo implantation, recent advances have identified various molecules relevant to implantation and uterine receptivity, including specific cytokines, chemokines, growth factors, adhesion molecules, lipid mediators, homeobox transcription factors, and ovarian steroid hormones[2,3,4]. However, it is still unknown whether they work independently, in parallel, or converge on a common signaling pathway. The mechanisms underlying endometrial receptivity and implantation remain unclear.

## Leukemia Inhibitory Factor (LIF)

The leukemia inhibitory factor (LIF) is a pleiotropic cytokine of the IL-6 family. Although named for its ability to inhibit proliferation of the mouse myeloid leukemic cell line M1 by inducing differentiation, LIF is able to exert various effects on a wide variety of tissues and cell types throughout the body. Stewart et al.[5] showed that female mice lacking a functional LIF gene are fertile, but their blastocysts fail to implant. However, the blastocysts are viable and when transferred to wild-type pseudopregnant recipients, they can implant, indicating the essential role of LIF in implantation. LIF is expressed in mouse uterine endometrial glands specifically on day 4 before implantation, and then in stromal cells surrounding the active blastocyst on day 5 during attachment reaction, suggesting the importance of LIF in uterine preparation and subsequently in blastocyst attachment[6]. The work has been extended and LIF has been shown to act at different levels of the reproductive axis[7]. LIF functions through its receptor (LIFR), which is composed of two subunits: the specific LIFRβ and gp130. The IL-6 family cytokines share gp130 as a common receptor subunit for signal transduction. Inactivation of gp130 also results in implantation failure[8]. LIF binds to LIFRβ, induces heterodimerization of LIFRβ and gp130, and then activates various signaling pathways, including JAK/STAT, MAPK, and PI3K/AKT. As a negative feedback loop, LIF is negatively regulated by suppressors of cytokine signaling (SOCS)[7].

## Calcitonin (CT)

Calcitonin (CT), a peptide hormone known to regulate calcium homeostasis, is a critical paracrine regulator of implantation. Under the control of progesterone, CT is shown to be expressed specifically in the rat and human endometrial glandular epithelial cells at the time of implantation[9,10]. Attenuation of uterine CT gene expression by antisense oligodeoxynucleotides results in a severe impairment of implantation[11]. CT functions through its cell surface receptor (CTR). CT down-regulates E-cadherin expression in the rodent endometrial epithelium during implantation[12], which may remodel adherens junctions between epithelial cells and thereby facilitate blastocyst implantation. Calcium signaling cascades are important in CT regulation of E-cadherin expression. The cytosolic calcium level rises rapidly in epithelial cells after CT stimulation, and calcium-dependent protein kinase C is also activated[12,13].

## Homeobox A10 (HOXA10)

The homeobox genes are a large and diverse group of genes characterized by the possession of a particular conserved DNA sequence, the homeobox, which encodes transcription factors with important roles in embryonic patterning and cell differentiation[14,15]. Some homeobox genes are important for human uterine development and receptivity. Along the developing paramesonephric duct, there is a differential pattern of homeobox gene expression. Four genes (HOXA9, HOXA10, HOXA11, and HOXA13) are involved, each corresponding to a region that will develop into a unique structure of the reproductive tract. Specifically, HOXA10 is expressed in the developing uterus during embryogenesis[16]. In adults, HOXA10 is expressed in the endometrial glands and stroma in a menstrual cycle–dependent manner, and its level rises dramatically in the mid-secretory phase. The expression persists in the decidua[16]. This expression pattern suggests that HOXA10 is necessary for uterine receptivity and decidualization. The evidence that HOXA10 is required for implantation comes from studies in knockout mice[16]. HOXA10 is regulated by ovarian steroid hormones[16]. In turn, HOXA10 is likely to mediate the effects of estrogen and progesterone by activation or repression of the endometrial downstream target genes involved in the implantation process. Some transcriptional targets of HOXA10 have been identified, including $\beta_3$-integrin subunit[17], EMX2[18,19], $GABA_A$ $\pi$ receptor[20], and Kruppel-like factor 9 (KLF9)[21].

## IMPLANTATION AND INFLAMMATION

A close similarity between embryo implantation and leukocyte extravasation has been noticed before. There are some similar molecular players, such as L-selectin and integrin, in these two different physiological processes[22]. It is also observed that local injury to the endometrium, such as endometrial biopsy taken during IVF/ET treatment, results in increased success of implantation, which is likely secondary to the development of an injury-induced inflammatory reaction at the fetal-maternal interface[23]. All these suggest the correlation between implantation and inflammation.

Although the specific action and mechanism of the inflammatory response in implantation have not been clearly understood, they are related to the activation of the immune system, characterized by increased activity of diverse innate immune cells and increased levels of inflammatory cytokines. At the time of implantation, under the stimulation of ovarian steroid hormones, the uterine environment is rich in inflammatory cytokines that recruit and activate specific innate immune cells. In turn, these immune cells enhance the endometrial expression of essential molecules that facilitate implantation[24]. Colony stimulating factor-1 (CSF-1) and several chemokines are identified to be the inflammatory cytokines important for leukocyte recruitment to the endometrium during implantation[25]. In both humans and rodents, the fetal-maternal interface is dynamically populated with a wide variety of innate immune cells. Of them, 65–70% are uterine-specific natural killer cells (uNKs) and 10–20% are antigen-presenting cells (APCs), including macrophages and dendritic cells (DCs). uNKs, of the $CD56^{bright}CD16^{-}$ NK subset, are shown to be important in directing trophoblast invasion by production of IL-8 and interferon-inducible protein-10 chemokines. Furthermore, uNKs secrete an array of angiogenic factors and induce vascular growth in the decidua. These functions are regulated by specific interactions of activating or inhibiting receptors on uNKs, and their ligands expressed on invasive trophoblasts or decidual cells[26]. DCs, which represent around 5–10% of the leukocyte population in the endometrium, appear to govern uterine receptivity by regulating tissue remodeling and angiogenesis through their synthesis of soluble vascular endothelial growth factor receptor 1 (sFlt1) and transforming growth factor-β1 (TGF-β1)[27,28].

## RESOLUTION OF INFLAMMATION

Inflammation serves as a protective response against injury and infection, while, if poorly controlled, it may develop to chronic inflammation with possible further tissue damage. Hence, inflammation is a two-edged sword. Over the years, how inflammation starts and the corresponding molecular mechanisms have been studied in detail, but little is known about how to switch it off. More and more studies show that resolution of inflammation is essential for the return of tissues to homeostasis and has become a new focus of inflammation research.

Resolution of inflammation is an active and preprogrammed process. It has been shown that the same mechanisms that induce inflammation also program its resolution, switching the production of lipid mediators from proinflammatory to proresolving[29,30]. During the initial steps of acute inflammation, eicosanoids — including prostaglandins (PGs) and leukotrienes (LTs) — regulate the early events, such as promoting vasodilatation, which facilitates the recruitment of polymorphonuclear cells (PMNs) into the tissue. As the inflammation progresses, PGs signal the end by activating the transcriptional regulation of

15-lipoxygenase (15-LO) in neutrophils, which in turn leads to the production of lipoxins from arachidonic acid (AA), and the more recently discovered resolvins and protectins[31]. These exert anti-inflammatory and proresolving effects via reducing vascular permeability, inhibiting PMN infiltration, as well as stimulating the recruitment of monocytes and the phagocytosis of apoptotic cells by macrophages.

## LIPOXINS

Lipoxins (LXs) are the first recognized lipid mediators with specific anti-inflammatory and proresolving effects.

Lipoxin $A_4$ ($LXA_4$) and its positional isomer lipoxin $B_4$ ($LXB_4$) are the major LXs in mammals. LXs are generated from AA by the sequential action of lipoxygenase (LO), mainly via transcellular biosynthesis involving the interactions of leukocytes, platelets, and epithelial cells in the context of inflammation. Leukocyte/platelet interaction is an important pattern. Two enzymes, 5-LO and 12-LO, are involved in this process. Leukocytes initiate the oxygenation of AA by 5-LO to release leukotriene A4 ($LTA_4$), which is then taken up and converted to $LXA_4$ by 12-LO in platelets. Interestingly, we recently found that platelet-derived microparticles can transfer 12-LO to leukotriene-producing mast cells, revealing a new pathway for $LXA_4$ biosynthesis[32]. LX generation can also be triggered by aspirin, which acetylates cyclooxygenase-2 (COX-2) and thus changes its catalytic activity into an R-LO. Accordingly, LXs synthesized in this route are 15R-epimers of LXs, specifically 15-epi-$LXA_4$ or 15-epi-$LXB_4$, carrying their C-15 alcohol group in the R configuration[33,34]. Native LXs undergo rapid inactivation *in vivo*, primarily via dehydrogenation at C-15 and ω-oxidation at C-20[34]. Thus, metabolically more stable analogues have been designed to resist degradation and retain their biological activity, holding potential as therapeutics.

LXs display selective actions on leukocytes. They are stop signals for PMNs, blocking their further infiltration into sites of inflammation by reducing vascular permeability. By contrast, they stimulate monocyte recruitment and the phagocytosis of apoptotic PMNs by monocyte-derived macrophages, which is also associated with a shift from release of proinflammatory to anti-inflammatory cytokines[33,34]. They also modulate the expression of genes involved in resolution and regulate NF-κB activation[34]. $LXA_4$, aspirin-triggered 15-epi-$LXA_4$, and their stable analogues, act with the LX-specific G protein-coupled receptor $LXA_4$ receptor (ALXR). Although $LXA_4$ and $LXB_4$ share biological activities, $LXB_4$ does not bind the ALXR. Functional studies have indicated the existence of a receptor that is activated by $LXB_4$, but this receptor has not been cloned[34]. Human ALXR is first identified in neutrophils and also expressed in several other types of leukocytes, bronchial epithelial cells, intestinal epithelial cells, and renal mesangial cells[35]. The expression of ALXR is transcriptionally up-regulated by various cytokines and responsible for the specific actions of LXs in resolution. Additionally, the actions of LXs are also mediated through antagonism of the cysteinyl peptide-LT receptor (CysLT), cross-talk with the growth factor receptor, as well as the nuclear receptor in DCs[35]. For example, LXs are proven to be potent inhibitors in angiogenesis induced by vascular endothelial growth factor (VEGF)[36,37,38,39,40]. ATL-1, an analogue of aspirin-triggered $LXA_4$, has been shown to regulate VEGF receptor-2 (VEGFR-2) phosphorylation in endothelial cells[38].

## LIPOXINS: A NOVEL REGULATOR IN EMBRYO IMPLANTATION

LX, derived from AA mainly via transcellular biosynthesis in the context of inflammation, is a novel proresolving lipid mediator and is thought to be the “stop signal” for inflammation. As discussed earlier, there is a close relationship between implantation and inflammation, so we are interested in the potential role of LXs in implantation.

## Supporting Evidence

- LX receptors on the endometrial epithelium

Implicit in the role of LXs during implantation is the presence of specific receptors for LXs in the endometrial epithelium. Significantly higher expression of ALXR was detected in endometrium in normal female rats under physiological conditions[41], suggesting possible involvement of $LXA_4$ in endometrial receptivity and implantation.

- The proresolution actions of LXs in the endometrium

The possibility that LXs have an inhibitory effect on blastocyst implantation could be further supported by the proresolution actions of LXs in the endometrium. Recent studies showed that $LXA_4$ may inhibit the progression of endometriosis[42,43], the pathogenesis of which is associated with inflammation. Inflammatory cytokines in the peritoneal microenvironment may trigger a cascade of events that eventually result in development and further progression of endometriosis.

## Possible Mechanisms

- LXs regulate the function of endometrial glands

Endometrial glands and their secretions are required for implantation[44]. Thus, it is proposed that LXs may suppress the expression of LIF and CT, two endometrial gland secretory molecules essential for implantation. If this hypothesis is true, the further question is: What is the specific molecular mechanism through which LXs regulate the function of endometrial glands?

- Neuroregulatory mechanism of LXs in the endometrium

The endometrium is a dynamic tissue that undergoes physiologic and characteristic morphologic changes during the menstrual cycle as a result of the effect of sex steroid hormones coordinately produced in the ovary. Under the influence of estrogen, there is an extremely rapid growth of both glands and stroma accompanied by angiogenesis. New genetic studies suggest that angiogenesis and neurogenesis are interdependent. Blood vessels and nerves use common signals and genetic pathways to regulate their development and function[45]. These findings, by inference, indicate the existence of a neuroregulatory mechanism in the endometrium. The identification of a novel subunit class of $GABA_A$ receptor termed $\pi$ in the endometrium provides a strong evidence for this inference[46]. The $GABA_A\pi$ receptor is important for implantation. As a target gene of HOXA10, the expression up-regulates during the window of implantation in the endometrium[20]. LX is proven to be a potent modulator in angiogenesis[36,37,38,39,40]; thus it is likely to be involved in the neuroregulation of the endometrium. LXs might regulate the endometrial $GABA_A\pi$ receptor expression via HOXA10, and consequently control the endometrial gland secretion of LIF and CT in the process of implantation.

Although the specific mechanism remains unclear, we think highly of the promising use of LXs as a novel contraceptive.

## ACKNOWLEDGMENTS

This work was supported by the Independent and Innovation Fund of Huazhong University of Science and Technology (20102D016).
